# Data Visualization and Analysis in Second Language Research

**DOI:** 10.3389/fpsyg.2022.833825

**Published:** 2022-04-06

**Authors:** Yiou Sun, Ping Wang

**Affiliations:** ^1^School of Foreign Language Studies, Jiangsu University of Science and Technology, Zhenjiang, China; ^2^School of Languages, Literacies and Translation, Universiti Sains Malaysia, Penang, Malaysia

**Keywords:** statistics, second language acquisition (SLA), data visualization, data analysis, R

## Abstract

I am working as both a TEFL teacher and an SLA researcher in China, doing SLA research. Recently, I have been working on new approaches to data analysis and I’ve found that a book titled “Data Visualization and Analysis in Second Language Research” by Dr. Guilherme D. Garcia is of great significance in empirical research in the field of SLA. This book serves only as a practical and user-friendly guide to beginners involved in SLA research, but also navigation to veteran SLA researchers devoted to new perspectives in data analysis. So far, the author has made his first attempt to connect data visualization under R to SLA research. The author reviews the previous research results and suggests some modifications through running R. From my perspective, it fills a gap in the SLA research practice—to lead readers into a new track of visualizing their reported results. Overall, to make an increasingly higher demand for SLA research, this book is particularly designated for quantitative data analysis in SLA. It is strongly recommended to write a book review to introduce readers to the realm of data visualization and help most SLA researchers better understand the potential value of R and visualization in SLA research.

*Data Visualization and Analysis in Second Language Research* opens a new door to researchers and TEFL teachers in Second Language Acquisition (SLA). Through the medium of the R language, the ultimate goal of this book is to promote a better understanding of published figures or reported results in the field of SLA. To date, R has been identified as one of the most novel and practical techniques for illustrating and displaying data in some social science research. As data visualization has been applied to social research, Guilherme D. Garcia intends to lead most SLA researchers and TEFL teachers into a new field of quantitative data analysis. Throughout the book, Garcia constitutes a noteworthy attempt at pointing out limitations of traditional statistical tools and offering more intuitive ways of displaying data through R to meet increasingly higher demand for SLA research. In general, the book succeeds not only in enabling veteran researchers to continue their exploration for an avant-garde method of analysis but ensuring its accessibility for novices as well.

The book is mainly written primarily for teachers or researchers of SLA. This step-by-step guide brings intended readers into the realm of visualization. The book consists of 10 chapters, falling into three parts (each with a brief introduction), an extensive glossary of key terms, a list of references for the whole volume, and an index (with glossary terms in bold). With scripts and figures per chapter, the book is more generously illustrated than other handbooks in the field.

The first part, part 1—Getting Ready (Chs. 1–2), namely the prep step to data visualization, involves terms and basic concepts in data analysis and statistical work. It helps readers of less proficiency in Statistics better understand the importance of reliable “data observed” in reality, the predictable patterns in unobserved data, and the evaluation of “empirical evidence” (p. 3). Also, the author provides a more robust statistical toolkit to achieve visualization in data analysis, which helps researchers transfer from effect size to *P*-values.

Chapter 1 introduces the key concepts in basic statistics: *p*-Values, Effect Sizes, Confidence Intervals, and Standard Errors, which lay a solid foundation for beginners and highflyers to follow the line of thoughts in this book. Chapter 2 touches upon limitations of the SPSS package: the enormous tables of larger sampling size leading to less effective communication of research in reported results; lack of stable analytical frameworks; firm reliance on observed data; risk of failing to help the reader understand “thinking process,” “narrative,” and “statistical results”; and “less variety in multitasking manipulation in more groups and lower degree of complexity in data analysis” (p. 6). The chapter unfolds as a richly exemplified argument that “R for data analysis is a smart decision along with convincing reasons, an open-resource with a substantial online community, a collection of tools for specific goals, a model of a more substantial power, faster speed than other packages and more effortless reproduction” (p. 15). Overall, the two chapters convince us of the visible advantage of R, leading us to an easy road to data visualization.

The second part, composed of chapters 3, 4, and 5, provides a roadmap for more accessible visualization techniques. This part is friendly to beginners who want to get into a new statistical analysis without starting from scratch. Chapter 3 focuses on continuous variables and investigates different plotting techniques to “visualize continuous data by using R—more specifically, the ggplot2 package” (p. 64). In this chapter, some basic principles are specified for better visualization, and the most common plots for continuous variables using ggplot2 are offered. Compared with the standard statistics package, R shows the same data, and the patterns it displays are not contradictory. They are simply two different perspectives of reporting the same variables and effects. Chapter 4 saves us the trouble of computing for “binary response variable,” which allows us to “transform categorical variables into continuous variables (counts or percentages)” (p. 86). In this chapter, the author exemplifies that “visualizing ordinal data is not so different from visualizing binary data” (p. 97). This invariably convinces us that data analysis through R is far more effective than the standard method and offers us a simplified way of calculating percentages in Binary data. Chapter 5 is the last but not the least in this part. It reminds us of “aesthetic characteristics such as font size, label orders” (p. 99). In doing that, the author introduces several aesthetic modifications for clarity and objectivity. To achieve better visualization, ggplot2 offers much flexibility: readers can create virtually any static figure as needed; readers have total control over the aesthetic parameters involved; readers could keep existing ggplot2 code for use without starting from scratch.

The third part—part 3 (Chs. 6–10) concentrates on the “regression model.” This part offers a variety of regression models for simple yet visualized regression in SLA. Chapter 6 reviews the definition of linear models and their applications in this field. It presents us with scripts with different examples. Following the instructions for operation, readers will get a high picture of essential steps in use: how to run them in R, how to interpret them, and how to report their results.

Chapter 7 continues with the regression model and sheds light on the limitation of “ANOVAs for categorical response variables” and offers a better solution to “binary response variables,” namely logistic regression (p. 143). In this chapter, the author leads us to the first review of some basics (Ch. 7.1) and then shows us how to run and interpret logistic models in R (Ch. 7.2). Chapter 8 offers a detailed analysis of ordinal models and centers on analyzing the last type of data—“ordinal variables”—tested in Likert scales. To illustrate data of two-opposite-ends and neutral labels of distinction, the author adopts ordinal models into analysis and pays fuller attention to logit ordered models, which help assist the process. The deeper the understanding of the model and its output, the closer the data being modeled is to the results reported. Chapter 9 explores hierarchical models and their advantages. This chapter thoroughly discusses three types of linear models and displays how hierarchical linear models can be used in logistic and ordinal models in chapters 7 and 8. In SLA research, one of the distinct advantages of the hierarchical model is that it is more effective and inclusive than non-hierarchical ones in dealing with “grouped factors” in analyses. Another distinctive feature of the hierarchical model is that running these models in R is straightforward though potentially complicated. On top of that, random effects could be an easier task in this model and adequately handled. Chapter 10 is the stepping stone to a more complex model, Bayesian statistics, a different outlook to data analysis.

The principal value of this book is to enable the author to call for a shift of focus from *t*-test and ANOVAs to R in the field of L2. Some classic works on data analysis, such as *A guide to doing statistics in second language research using SPSS* (Larson-Hall, 2011), present numerous examples of performing statistics with some general preference-based principles for constructing and assessing graphs through SPSS. In contrast to traditional statistics, *Data Visualization A PRACTICAL INTRODUCTION* (Healy, 2019) provides a clear guide to R in data analysis. The book is recommended to help the readers quickly look at data and present results by running R to “make plots in a well-informed way, specify the relationship between variables and visible elements, and build up images layer by layer.” It is indeed a practical guide to data visualization under R but fails to attract a much wider potential readership by working at the application of R in a broader range of social science research, such as SLA, an essential branch of applied linguistics. By contrast, more recently published books, such as Statistics for Linguistics with R: A Practical Introduction (Gries, 2013) and *Statistics for Linguists: An Introduction Using R* (Winter, 2019), function as a practical guide to statistics in linguistics. They are more of handbooks of exemplifying R in statistics for linguistics in general rather than providing concrete examples of using R in better solutions to problems or clearly illustrated figures with L2. This quick solution is urgently needed in L2 research regarding statistical methods and reporting results.

All books mentioned above fail to discuss the limitations of traditional statistics, showing that means, medians, and standard deviations might indicate problems with the testing hypothesis in various groups. To further investigate the R in SLA to improve the accuracy of the final empirical research, Garcia demystifies the workings of R and provides better visualization of data. More importantly, Garcia (2021) makes up for the gaps between data visualization and SLA research. Looking closely at how R works to show more details with fuller evidence in the statistics, the author finds that R language not only connects the SPSS with other statistical models but explores the interface between SPSS and R as well, which helps readers to gain a comprehensive picture of how is visualization achieved in running ggplot. Some case studies show that data visualization is better output in reporting results. For example, Russell and Spada (2006), Lyster and Saito (2010), and Loewen (2012) published comprehensive reviews on the role of feedback and investigated the effect of different types of corrective feedback on English learning.

Among all the articles mentioned above, Lyster and Saito focus on meta-analysis and present an overview of the 15 studies with information concerning the number of participants, their age and L1 background, linguistic targets, CF types, treatment length, and types of outcome measures. Results shown in the line graph indicate that “explicit correction did not prove significantly different from either recast or prompts—neither in between- nor within-group contrasts” (Lyster and Saito, 2010). Garcia reconfirms the theoretical study with R that R could illustrate every point more accurately and demonstrate the details with a higher level of visualization and accuracy. He further argues that “box plots are underrated and underused in second language research” (p. 75). The data analysis undergoes three individual stages shown in Garcia (2021) (pp. 76–80). It is generally detected that R makes a difference to the published results employing traditional statistical methods. The first figure provides a very informative picture of the datasets. The semitransparent data points on the background help the reader access the actual data points. As stated in the principle of R, “the more two box plots overlap, the less likely they are statistically distinct” (p. 76). For example, if readers inspect the facet for participants, they will notice that box plots for Explicit correction and Recast overlap almost the same. As a result, these participants do not seem to be affected by feedback the same way as the other two groups. This bar plot coincided with the results by Lyster in 2010. The second figure help readers look closely at the standard error bars, which are very small. It is evident that there is minimal overlap between explicit instruction and recast. Readers may assume that the effect of feedback is of no correlation with box plots. If researchers had displayed them in the bar plots, on the other hand, they would less likely fall into the illusion that feedback has no effect, given that the error bars do not overlap in most facets. Looking more closely at two means of reporting results, readers might be thinking about making a serious attempt at combining a box plot information and the mean and standard error of a bar plot with error bars. In the last stage, the author achieves both. Box plots also display means and common errors, which other researchers failed to observe in previous research. In summary, R provides researchers with more options for sampling and grouping data and more alternatives to simplify the data reporting with a higher aesthetically effect for the sake of accuracy and clarity.

This volume is a good resource of methods in data analysis and an easy-to-understand textbook with samples, scripts and exercises at the end of each chapter. To introduce the beginner to the R and its primary functions behind the output, the author takes much copy in different chapters to review the basic concepts and some essential issues in manipulations of both SPSS and R. This step paves the way for a better understanding of new statistical models in SLA research. It undeniably captures the reader’s attention by using R to visualize reported results in data analysis. It paints a detailed picture of the statistical models as almost every chapter in part 2 and part 3 adds a new model and turns to a different statistical model. It also provides a solid orientation to fundamental knowledge in statistics by presenting the key issues and concepts in the last chapter and making a smooth transition between the old model and the new ones.

Moreover, although basic knowledge of statistics is not fully expounded, Garcia’s introductory chapters and section prefaces the glossary, exercises, summaries, and notes on further reading, along with many of the chapters, are invaluable for newcomers to visualization. Another minor advantage of this handbook that cannot be overlooked is that it illustrates R language and its primary functions used in data visualization and compares some of the limitations of SPSS and other Statistical packages in “grouped variables” with merits new statistical models. The comparison part points out that great potential data visualization in SLA research. Many chapters (Chs. 6–9) demonstrate concrete samples and operations of R and expound on the practicalities of each model and their limits, coming up with sophisticated solutions to data analysis, and it is of great value for researchers in SLA.

While the handbook presents an extensive range of methods of running R and many applicable analytical models from various practical perspectives, some areas are yet to receive feedback from SLA researchers. The book has a rich collection of various statistic methods under R but a limited number of case studies in SLA. It may also expose readers in the field of SLA to some of its weaker aspects in the actual application. A large number of chapters included limits their length, potentially explaining why R, compared with other statistical methods, makes a relatively small number of contributions to current issues concerning SLA. The visualization effect does not go far beyond the ken of average SLA researchers by giving examples of why visualization matters in data reporting that illustrates data of significance to the research rather than seeking aesthetic value and superficially formed modifications. Lack of space may be the reason why the volume hints at solutions for overcoming the limitations of SPSS, rather than providing more examples shown in chapters. For instance, in chapter 10, the author follows every step toward avoiding being trapped in a comfort zone, in which SLA researchers are more satisfied with “simplistic understanding of statistical inference” (p. 235) rather than with further explorations in more complex issues or in-depth interpretations. Compared with Frequentist, running models under R, Bayesian, for example, demands more knowledge or expertise in computer programming, which most TEFL teachers or practitioners lack in their knowledge bank. Second, in the field of SLA, Bayesian analysis remains a newly emerging field to most reviewers or readers working on L2, many of whom get accustomed top-values and find it tough to push themselves out of their comfort zone. Most SLA researchers are less likely to go beyond the apparent and comforting SPSS or other statistics unless R has proven more effective and straightforward in dealing with results and analysis. On the other hand, due to the limitation of the author, examples illustrated in the SLA field covers a limited range of L2 study, and many chapters only touch upon issues of L2 grammar, L2 pronunciation, and correct feedback, being a small part of contributions that R makes to SLA. These uncovered areas evidence the shifting ground that SLA research develops with R as it deals with constant transformations in the roles that different modes and their relations play in ever-changing SLA practices.

Despite these minor shortcomings, the arrangement of the book is so user-friendly that each code block in the R language is carefully examined. As a valuable statistical computing tool, R allows researchers to easily access data and reproduce code blocks in previous models. To the best of luck, R is open-source and can be widely used in nearly every field of social research, from which we SLA practitioners have thousands of packages to choose. Packages like “tidyverse” and “brms” allow more and more SLA teachers and researchers to complete complicated projects by running a few code blocks in R. The author, as a result of this, strongly recommends SLA researchers to read over this navigation to data visualization. This roadmap not only serves as a practical guide to data analysis in SLA but leads you to the right track of researching language acquisition as well. With great expectation, the author is looking forward to an increasing number of new perspectives emerging from data visualization and analysis, enriching language teaching and learning research.

## Author Contributions

YS wrote the first draft of the manuscript. YS and PW contributed to manuscript revision, read, and approved the submitted version. Both authors contributed to the article and approved the submitted version.

## Conflict of Interest

The authors declare that the research was conducted in the absence of any commercial or financial relationships that could be construed as a potential conflict of interest.

## Publisher’s Note

All claims expressed in this article are solely those of the authors and do not necessarily represent those of their affiliated organizations, or those of the publisher, the editors and the reviewers. Any product that may be evaluated in this article, or claim that may be made by its manufacturer, is not guaranteed or endorsed by the publisher.
